# Immune checkpoint blockade in hematological malignancies: current state and future potential

**DOI:** 10.3389/fonc.2024.1323914

**Published:** 2024-01-23

**Authors:** Prateek Pophali, Juan Carlos Varela, Jacalyn Rosenblatt

**Affiliations:** ^1^Division of Hematology and Hematological Malignancies, Beth Israel Deaconess Medical Center, Harvard Medical School, Boston, MA, United States; ^2^Division of Hematology and Oncology, Orlando Health Regional Medical Center, Orlando, FL, United States

**Keywords:** immune checkpoint inhibitors, hematological malignancies, PD-1, CTLA-4, TIGIT, TIM-3, LAG-3, CD47

## Abstract

Malignant cells are known to evade immune surveillance by engaging immune checkpoints which are negative regulators of the immune system. By restoring the T-lymphocyte mediated anti-tumor effect, immune checkpoint inhibitors (ICI) have revolutionized the treatment of solid tumors but have met rather modest success in hematological malignancies. Currently, the only FDA approved indications for ICI therapy are in classic hodgkin lymphoma and primary mediastinal B cell lymphoma. Multiple clinical trials have assessed ICI therapy alone and in combination with standard of care treatments in other lymphomas, plasma cell neoplasms and myeloid neoplasms but were noted to have limited efficacy. These trials mostly focused on PD-1/PDL-1 and CTLA-4 inhibitors. Recently, there has been an effort to target other T-lymphocyte checkpoints like LAG-3, TIM-3, TIGIT along with improving strategies of PD-1/PDL-1 and CTLA-4 inhibition. Drugs targeting the macrophage checkpoint, CD47, are also being tested. Long term safety and efficacy data from these ongoing studies are eagerly awaited. In this comprehensive review, we discuss the mechanism of immune checkpoint inhibitors, the key takeaways from the reported results of completed and ongoing studies of these therapies in the context of hematological malignancies.

## 1 Introduction

Advances in cancer immunotherapy has significantly changed the treatment landscape for the treatment of hematological malignancies. The graft versus leukemia effect in the setting of allogeneic transplantation was the earliest demonstration of the potency of the immune system in targeting malignancy (1). A major area of research interest has focused on harnessing the power of the immune system to more specifically target the malignant clone, in an attempt to capitalize on the unique potency of immune mediating killing, while minimizing toxicity. A critical milestone in the history of cancer immunology was the discovery of immune ‘checkpoints’, which act as negative regulators of effector T cells. Biologically, these immune checkpoints serve the important function of self-tolerance to avoid auto-immunity and are expressed on immunosuppressive regulatory T cells. Activation of these checkpoints result in decreased activation, proliferation, cytokine production and ultimately an apoptotic fate of the effector immune cells. Malignant cells hijack these negative regulators of the immune system to evade immune mediated killing. Inhibiting these immune check points with monoclonal antibodies has been shown to be a successful therapeutic strategy leading to significant improvement in the response and survival rates in certain malignancies. Despite revolutionizing the treatment landscape of multiple solid tumors including kidney cancer, melanoma and lung cancer, immune checkpoint inhibitors (ICI) have met mixed success in the treatment of hematological malignancies. Numerous PD-1 axis and CTLA-4 inhibitors are now commercially available and multiple other immune checkpoint targeting therapies are in development. This review will explore the various targetable immune checkpoint pathways and the status of their clinical application in the context of hematological malignancies.

## 2 Immune effector cells and their checkpoints

Antigenic stimulation of the T cell receptor (TCR), a clone specific cell surface complex, is a crucial step towards activation of the T cells (2). However, an additional co-stimulation step is required for T cell activation and proliferation. CD28 protein and its family members were noted to be potent co-stimulatory molecules for T cell activation (3, 4). CD28 ligands B7-1 (CD80) and B7-2 (CD86) are found on antigen presenting cells and binding of CD28 with B7-1/2 leads to activation of the co-stimulatory domain CD28.

Cytotoxic T lymphocyte associated antigen 4 (CTLA-4) is structurally similar but performs the opposite function to CD28. On binding to the same ligands, B7-1/2, expressed on the antigen presenting cells (APC). CTLA-4 causes inhibition of T cell activation primarily by interfering with the CD28-B7-1/2 interaction. Along with directly competing with CD28 for binding its ligands, it also internalizes the ligands leading to decreased binding with CD28 thereby decreased secretion of stimulatory cytokines like IL-2 and causes reduced T cell proliferation (5, 6). CTLA-4 also activates SHP2 and PP2A phosphatases which prevent phosphorylation of CD3ζ an important part of the TCR complex (7). Presence of CTLA-4 on immunosuppressive regulatory T cells further confirmed its role as a negative regulator of the immune system. As such, inhibition of CTLA-4 to elicit a therapeutic immune response against cancer cells was a highly attractive proposition. This was successfully demonstrated by Allison et al. in 1996 in a mouse model. They showed that neutralizing anti-CTLA-4 antibodies lead to eradication of colon cancer and fibrosarcoma as well as induced anti-tumoral immunity (8). After demonstrating success in clinical studies, Ipilimumab was the first CTLA-4 monoclonal antibody to get FDA approval in 2011 (9).

The programmed cell death protein (PD)-1 was identified to play an analogous role to CTLA-4 and was noted to be expressed on the T cells after TCR stimulation. On binding to its ligands PDL-1 (also known as B7-H1) and PDL-2 (B7-DC), it induces inhibitory intracellular signaling and inactivation of T cells (10). Similar to CTLA-4, PD-1 activates the SHP1 and SHP2 phosphatases which inactivate downstream signaling needed for T cell activation (11, 12). Contrary to regulation of early T cell activation in the lymphoid tissues by CTLA-4, the PD-1 axis is thought to play an important role in continued activation of T cells especially in the peripheral tissues and on engagement with the ligands, PD-1 induces a dysfunctional T cell state known as “T cell exhaustion” (13). Tumor cells use this negative regulator of the effector T cells by overexpressing PDL-1 or PDL-2. Subsequently this PD-1 axis was explored to be a therapeutic target to restore immune cell cytotoxicity against cancer cells. Monoclonal antibodies against PD-1 and PDL-1 have shown remarkable anti-tumor activity and have led to meaningful improvement in the clinical outcomes in various cancers.

Lymphocyte activation gene-3 (LAG-3, also known as CD223), another immune checkpoint, is a transmembrane protein expressed on the surface of both effector and regulatory T lymphocytes after antigen mediated stimulation (14). LAG-3 binds with stable complexed of peptide and MHC-II on the antigen presenting cells. Initial thought was that LAG-3 competitively inhibits CD4-MHCII binding but recently it was shown that LAG-3 suppressed T cells by transducing inhibitory signaling via in the intracellular domain (15). It is unclear if MHC II is the sole functional ligand of LAG-3. Fibrinogen-like protein 1 (FGL1) secreted by the liver is also implicated to be an immune-inhibitory ligand of LAG-3 particularly affecting CD8+ T cells. High levels of FGL-1 expressed by cancer cells are associated with poor prognosis (16, 17). Given its role in immune tolerance for cancer cells, LAG-3 blockade is being explored as a therapeutic target.

T cell immunoglobulin and mucin domain 3 (TIM-3) is an inhibitory immune checkpoint receptor frequently co-expressed with PD-1 on tumor infiltrating T cells as well as other innate immune cells like monocyte/macrophages, NK cells and dendritic cells. Galectin-9 (GAL-9) or carcinoembryonic antigen related cell adhesion molecule (CEACAM1) are known ligands of TIM3 which cause inhibitory signaling downstream causing T cell exhaustion or apoptosis (18, 19). TIM-3 signaling has been proposed as a resistance mechanism for PD-1 directed therapy due to the noted co-expression and therefore has been explored as a therapeutic target in many malignancies including hematological malignancies (20, 21).

T cell immunoglobulin and ITIM domain (TIGIT, also known as Vstm3, VSIG9, WUCAM) is another co-inhibitory transmembrane molecule exclusively expressed on T cells including CD4+, CD8+, regulatory T cells and NK cells. TIGIT activates a complex immunoregulatory network on binding with its multiple ligands including CD155, CD112, CD113 and Nectin-4 expressed by antigen presenting cells as well as cancer cells. The primary high affinity ligand for TIGIT is thought to be CD155 (22, 23).. Although not completely understood, TIGIT causes T cell inhibition by multiple mechanisms including TCR downregulation, competing for ligands with an activating co-receptor CD226 as well as enhancing the immunosuppressive effects of regulatory T cells. Given its role in T cell inhibition and immune escape mechanism, TIGIT blockade has been explored as a therapeutic target in hematological malignancies (24). LAG-3, TIM-3 and TIGIT biology and its role for treatment of cancers has been reviewed in detail elsewhere (25, 26).

Macrophages are phagocytic cells and an integral part of the innate immune system. While these are traditionally thought to be functioning against infectious pathogens/foreign bodies, tumor associated macrophages were discovered to be involved in multiple malignancy processes like angiogenesis, metastasis, regulation of tumor microenvironment (27). CD47 is membrane protein first discovered to be present on erythrocyte surface. Senescent erythrocytes with diminished expression of CD47 are removed from circulation by macrophages in the spleen and liver (28). Normal erythroid cells are able to avoid this removal by expression of CD47 on their membrane which bind with macrophage inhibitory receptor signal regulatory protein alpha (SIRPα). This leads to downstream activation of SHP-1 and SHP-2 phosphatases causing phagocytic inhibition. Therefore, CD47 is commonly referred to as ‘do not eat me’ signal. Subsequently, it was discovered that CD47 is commonly over expressed on most tumor cells like AML, MDS, NHL as a macrophage checkpoint (29). Subsequently, CD47/SIRPα axis blockade was shown to effectively promote tumor cell phagocytosis as well as enhanced antigen presentation leading to a strong T cell mediated tumor response (30, 31). CD47 inhibition has progressed significantly in clinical investigation however a strong efficacy signal is yet to be noted.

## 3 Immune checkpoint blockade in lymphomas

### 3.1 Hodgkin lymphoma

Classic hodgkin lymphoma (cHL) has a unique biology. It is characterized by the malignant Hodgkin and Reed Sternberg (HRS) cells which comprise ~2% of tumor cells. The remaining tumor bed is primarily comprised of reactive T cells and immune cells. Studies have shown that these reactive immune cells have an exhausted phenotype leading to loss of effector function. This is due to overexpression of over-expression of PDL-1 and PDL-2 on the malignant HRS cells caused by amplification of the 9p24.1 locus (32). Treatment of cHL has evolved significantly over the years with combination chemotherapy as the backbone. Recently BV-AVD (Brentuximab vedotin, doxorubicin, vinblastine, dacarbazine) replaced ABVD (doxorubicin, bleomycin, vinblastine, dacarbazine) based on the improved survival with BV-AVD showed in the ECHELON-1 trial (33). Despite a large proportion of patients will achieve a complete response with frontline therapy, patients who have primary refractory or relapsed disease have poor outcomes. Salvage chemotherapy and high dose chemotherapy with autologous hematopoietic cell transplantation (auto-HCT) are used in this setting with significant treatment associated complications. Given the immunosuppressive biology of the HRS cells by overexpression of PDL-1/2, immune checkpoint blockade was first investigated in relapsed/refractory cHL. Checkmate-205 was a phase II trial that studied nivolumab a monoclonal PD-1 antibody in 243 patients with relapsed/refractory cHL after failure of autoHCT. The overall response rate (ORR) was 69% with complete response (CR) in 16% patients. Median time to response was ~2 months and patients achieved response for a median duration of 16.6 months (34). Pembrolizumab another monoclonal PD-1 antibody was also studied in Keynote-087. In this study, 210 patients with relapsed refractory cHL who had previously received at least 2 lines of therapy including autoHCT and/or brentuximab vedotin were treated with pembrolizumab monotherapy until disease progression or unacceptable toxicity. The ORR was 72% with a CR rate of 28%. Similar to checkmate-205, median duration of response was 16.6 months. Another noteworthy finding from Keynote-087 was that among patients in CR who had discontinued pembrolizumab and subsequently had disease progression, 74% had an objective response lasting for a median of ~15 months (35). In both trials with nivolumab and pembrolizumab, patients who were BV naïve, had improved responses. Subsequently, a phase III randomized controlled trial (RCT), Keynote-204, comparing pembrolizumab and BV in the relapsed/refractory setting showed improved median progression free survival (PFS) with pembrolizumab (13.2 months) as compared to BV (8.3 months) (p 0.0027) Tolerance to pembrolizumab was better as compared to BV (36). Pembrolizumab in combination with chemotherapy regimen GVD and nivolumab with ICE have both shown impressive responses (ORR 100%, CR ~90%) as first salvage therapy for R/R cHL (37, 38). Based on these data, both pembrolizumab and nivolumab have been approved for the treatment of relapsed/refractory cHL. PD-1 blockade has also been studied as post autoHCT consolidation in high-risk patients with relapsed cHL with improved PFS although mature data is awaited at this time (39, 40). PDL-1 monoclonal antibody, Avelumab was studied in a phase 1 trial (JAVELIN Hodgkin) in patients with relapsed/refractory cHL which showed an ORR of ~42% with CR in 19.4% patients (41). Along with combination of chemotherapy and PD-1 blockade, studies have also evaluated the efficacy of a chemotherapy sparing salvage regimen of BV+PD-1 blockade in relapsed cHL. The ORR was 85% with a CR rate of 67%; these were durable responses with 3 year PFS ~77% (42). Given the resounding success of PD-1 blockade in the relapsed/refractory setting, there is excitement about the role of PD-1 blockade in combination with chemotherapy in the frontline setting. Results from the southwest oncology group (SWOG) 1826, a phase III trial comparing nivolumab-AVD Nivolumab (N)-AVD vs BV-AVD were presented at the 2023 ASCO meeting. With approximately 480 patients in both arms, the 1-year PFS rate was superior with N-AVD as compared to BV-AVD (94% vs 86%; HR 0.48; p=0.0005) with lower rates of adverse effects in the N-AVD arm (43). Full publication from this trial is eagerly awaited and has the potential to change standard frontline therapy for cHL. See **Table 1** for key trials in hodgkin lymphoma.

**Table 1:** Key trials with Immune checkpoint inhibitors in Hodgkin lymphoma.

Trial	Phase	Intervention	Checkpoint target	Population	Efficacy	Adverse events (grade3-4) of checkpoint inhibitors
SWOG 1826 (NCT03907488)	III	Nivolumab-AVD vs Brentuximab vedotin (BV)-AVD	PD-1	Newly diagnosed advanced stage cHL (n=994)	1-year PFS: 94% vs 86% (HR 0.48; p=0.0005)	
CHECKMATE 205 (NCT02181738)	II	Nivolumab	PD-1	R/R cHL (n=243)	ORR 71% mPFS 15 months 5y OS 71%	Increased lipase (5%) Neutropenia (3%) Increased ALT (3%)
NCT02572167	I/II	Nivolumab+ BV	PD-1	R/R cHL (n=91)	ORR 85% 3-year PFS 77% 3-year OS 93%	Pneumonitis (3%) Rash (1%)
KEYNOTE 204	III	Pembrolizumab vs Brentuximab vedotin	PD-1	R/R cHL (n=304)	mPFS 13.2 vs 8.3 months (HR 0.65; p=0.0027	Pneumonitis (4%) Neutropenia (2%)
KEYNOTE 087 (NCT02453594)	II	Pembrolizumab	PD-1	R/R cHL (n=210)	ORR 71% mPFS 13.7 months	Total 13% Neutropenia (2.4%) Pericarditis (1%) Diarrhea (1%)
JAVELIN Hodgkins (NCT02603419)	I	Avelumab	PDL-1	R/R cHL (n=31)	ORR 42% mDOR 6.9%	Rash (7%) Increased lipase (7%) Penumonitis (3%) Immune thrombocytopenia (3%)

Given the synergy that the combination of PD-1/CTLA-4 blockade has demonstrated in some solid tumors, including melanoma and renal cell carcinoma, this combination is being studied in cHL. In a phase 1/2 trial, patients with relapsed/refractory cHL were treated with either combination of BV and nivolumab or ipilimumab (CTLA-4 antibody) or both. This study demonstrated improved responses with BV+ nivolumab over BV+ ipilimumab or BV+ both groups. Unfortunately, about 43% patients in the BV+ipilimumab group suffered grade 3-4 adverse events. The phase 2 study comparing BV+ nivolumab with or without ipilimumab is ongoing (NCT01896999) (44).

One approach to overcome resistance to treatment with PD-1 inhibition is to combine PD-1 blockade with inhibitors of other checkpoints co-expressed on exhausted T cells, such as LAG-3. Favezelimab (MK-4280), a humanized IgG4 LAG-3 inhibitor, was studied in combination with pembrolizumab in patients with relapsed/refractory cHL. The combination demonstrated an ORR of 73% with an impressive CR rate of 30%. Median PFS was 19.4 months. Common adverse reactions included infusion reaction and hypothyroidism (immune related adverse event). 23% patients had grade 3-4 adverse reactions (45).

### 3.2 Non-Hodgkin lymphoma

Non-Hodgkin lymphoma (NHL) is a heterogenous group of malignant lymphoproliferative disorders classified according to the cell of origin into B cell NHL (>85%) and the less common T/NK cell NHL. Pre-clinical studies have shown that intratumoral CD4+CD25+ regulatory T cells suppress the infiltration of effector T cells in B cell NHL leading to immunosuppressive tumor microenvironment (46). These cells are enriched in negative immune regulators like CTLA-4 and therefore CTLA-4 blockade with ipilimumab was studied in a phase 1 trial for patients with relapsed/refractory B cell NHL. Unfortunately, of the 18 patients treated only 2 patients had clinical responses (11%) (47). Subsequently, ipilimumab in combination with rituximab in patients with R/R B cell NHL showed a modest ORR of 24% with median PFS of 2.6 months (48). Combination of CTLA-4/PD-1 blockade with Ipilimumab/nivolumab showed ORR was 19% with median PFS of only 1 month (49).

PDL-1 expression on large B cell lymphoma cells was shown to have inferior outcomes mediated by suppression of anti-lymphoma immune response. As such, intercepting the PD-1/PDL-1 axis has been evaluated for clinical efficacy against R/R B cell NHL (50, 51). Multiple early phase studies showed safety but low efficacy of PD-1/PDL-1 blockade in R/R B cell NHL. In a multi-center phase 2 study of nivolumab in 121 patients with R/R DLBCL ineligible or refractory to autoHCT, the ORR was <10% (52). Similarly, in patients with R/R FL, the ORR of single agent nivolumab was a meager 4% (53). Another study in patients with FL (rituximab sensitive disease relapsed after >1 line of treatment), combination of pembrolizumab and rituximab had impressive ORR 67% with CR rate of 50%, however these were rituximab sensitive patients and it remains unclear if pembrolizumab had added benefit (54). A triplet combination of obinituzumab, lenalidomide and atezolizumab for patients with R/R FL showed an impressive CR rate of 72% with about 67% patients being refractory to rituximab and alkylator combination (55). However, durvalumab (anti PDL-1 antibody) in combination with Bruton Kinase inhibitor (BTKi) ibrutinib showed efficacy similar to single agent ibrutinib (ORR ~25%) in patients with R/R FL and DLBCL with added immune related toxicities (56). Addition of durvalumab to frontline R-CHOP in high risk DLBCL patients did not provide additional benefit (57). In patients with relapsed CLL or with Richter transformation (RT), pembrolizumab had a preferential efficacy in patients with RT (ORR 44%) while none in patients with CLL (58). Pembrolizumab in combination with dinaciclib (cyclin dependent kinase 9, CDK9 inhibitor) in patients with R/R CLL, DLBCL and multiple myeloma (MM) showed a ORR of 20-30% in patients with CLL and DLBCL whereas 0% in patients with myeloma (59). Post autoHCT consolidation with pembrolizumab in DLBCL did not show improvement in PFS (60). Pembrolizumab has also been studied in the context of post CD19 CAR-T relapse. The response rate noted was 25%. Correlative studies showed that there was increased in the CAR-T cells by mass cytometry by time of flight and increase in CAR19 transgene levels in patients those responded to pembrolizumab (61).

In contrast to other B cell NHL, PD-1/PDL-1 blockade in primary mediastinal B cell lymphoma (PMBCL) has shown robust activity and is FDA approved. PMBCL is common in adolescents and young adults. Although it is morphologically similar to large B cell lymphoma, genomically, PMBCL is closer to cHL frequently involving alterations of 9p24.1 resulting in over-expression of PDL-1/2. Unsurprisingly, early studies with pembrolizumab in R/R PMBCL showed ORR of 41% with durable responses (62). The final analysis of Keynote 170 again showed ORR of 41% with CR rate of ~21%. 4-year PFS in all patients was 33%. Impressively, all patients achieving CR had durable responses without relapses after 4 years of follow up (63). In combination with brentuximab vedotin, nivolumab showed an impressive ORR of 70% with CR rate of 43% in patients with CD30+ R/R PMBCL with median duration of response not reached after about 11 months of follow up (64). Please see **Table 2** for key trials in non hodgkin lymphoma.

**Table 2:** Key trials with Immune checkpoint inhibitors in Non-Hodgkin lymphoma.

Trial	Phase	Intervention	Checkpoint target	Population	Efficacy	Adverse events (grade3-4) of checkpoint inhibitors
NCT02038933	II	Nivolumab	PD-1	R/R DLBCL (n=121)	ORR 10% mPFS 1.9 months mOS 12.2 months	Neutropenia (4%) Thrombocytopenia (3%) Increased lipase (3%)
CHECKMATE-140 NCT02038946	II	Nivolumab	PD-1	R/R FL (n=92)	ORR 4% mPFS 2.2 months	Diarrhea (2%) Neutropenia (2%) Pneumonitis (1%)
NCT03003520	II	Durvalumab with R-CHOP or R^2^CHOP	PDL-1	Untreated high-risk DLBCL (n=46)	CR 54% PR 19%	
NCT02401048	I/II	Durvalumab with ibrutinib	PDL-1	R/R FL or DLBCL (n=61)	ORR 25% mPFS 4.6 months mOS18.1 months	Neutropenia (21%) Rash (7%) Pneumonitis (3%)
NCT02332980	II	Pembrolizumab	PD-1	CLL with RT (n=9) or R/R CLL (n=16)	ORR 44% for CLL with RT ORR 0% for R/R CLL	Hematological toxicity (20%) Rash (4%)
KEYNOTE-170 NCT02576990	II	Pembrolizumab	PD-1	R/R PMBCL (n=53)	ORR 41.5% mPFS 4.3 months 4-year PFS 33% mOS 22.3 months 4-year OS 45.3%	Neutropenia (13%) Increased AST (2%)
CHECKMATE-436 NCT02581631	II	Nivolumab with brentuximab vedotin	PD-1	R/R PMBCL (n=30)	ORR 73% mPFS NR mOS NR	Neutropenia (7%) Rash (3%) Colitis (3%) Immune mediated hepatitis (3%)
CITN-10 NCT02243579	II	Pembrolizumab	PD-1	R/R CTCL (n=24)	ORR 38%	Rash (17%) Arthritis (4%) Pneumonitis (4%) Colitis (4%) Immune mediated hepatitis (4%)
NCT03075553	II	Nivolumab	PD-1	R/R PTCL (n=12)	ORR 33% mPFS 2.7 months mOS 6.7 months	Hyperprogression (33%)
NCT02953509	I	Hu5F9-G4	CD47	R/R NHL (n=22)	ORR 50% CR 36%	Infections (18%) Anemia (4.5%) Infusion reaction (4.5%)
NCT03530683	I	TTI-662	CD47	R/R lymphomas (n=42)	ORR 9/27	Thrombocytopenia (5%) Neutropenia (9%) Anemia (2%)

The mechanism of resistance to PD-1 axis blockade in NHL is unclear. Proposed mechanisms include low lymphocyte infiltration of the tumor, low MHC expression on tumor cells, presence of immunosuppressive cells and up-regulation of other immune checkpoint proteins (65, 66). Other T cell checkpoints (LAG-3, TIM-3, TIGIT) have been associated with lymphoma progression but clinical data on therapeutic targeting of these checkpoints are not available yet. LAG-3 inhibition in combination with PD-1 axis blockade is being studied in R/R lymphomas in an effort to overcome resistance to PD-1 directed monotherapy and results are awaited (NCT02061761). TIM-3 has been noted to be overexpressed via IL-12 which results in T cell exhaustion in FL, however clinical data regarding safety and efficacy is not available yet (67). Similarly, a pre-clinical model showing combination of PD-1 and TIGIT blockade having synergistic effect in eliminating lymphoma cells has been reported (68).

It has been demonstrated that CD47 expressing lymphomas have poor outcomes and frequently leads to extra nodal dissemination of the malignant lymphoma cells. Therefore, targeting of CD47 or the anti-phagocytic ‘do not eat me’ signal presents an exciting therapeutic opportunity to evoke macrophage mediated immune response augmented by rituximab (69–71). Magrolimab (Hu5F9-G4) is a first in class, humanized IgG4 monoclonal antibody targeting CD47. In a phase 1 study in 22 patients with R/R NHL, magrolimab in combination with rituximab, 50% patients had an ORR while 36% having a CR. At last follow up, 91% of the responses were ongoing (72). Another CD47 inhibitor, TTI-622, is being evaluated in combination with rituximab for treatment of R/R NHL with ORR of 20-30% (73, 74).

### 3.3 T/NK cell NHL

T/NK cell NHL, a rare subtype of NHL, is often associated with poorer prognosis compared to it’s B cell counterpart. Cutaneous T cell lymphomas (CTCL) (eg. Sezary syndrome, mycoises fungoides) are considered indolent whereas peripheral mature T cell lymphoma (PTCL) an all-inclusive term for the rest of T cell lymphomas usually are aggressive. These are typically treated with combination chemotherapy. However, these therapies are not curative, and most patients will relapse. Immune checkpoint therapies targeting CTLA-4, PD-1 axis cause activation of the T cells, therefore there is a theoretical concern for stimulating growth of T cell lymphomas leading to hyper-progression. Accordingly, in a study with nivolumab in R/R PTCL, 4 of the 12 patients treated had hyper-progressive disease and the responses achieved were short lived. The study was discontinued (75). However, in a multicenter phase 2 study of pembrolizumab in 24 patients with R/R CTCL, the ORR was 38% with durable responses (>50 weeks). A transient worsening of erythroderma and pruritis occurred in ~50% patients followed by improvement; however hyper progression was not noted (76). Another study with avelumab (anti PDL-1 antibody) in R/R extra-nodal NK/T cell lymphoma (ENKTL) showed ORR of 38% with CR in 24% patients. Although the non-responders showed early progression, responders continued to receive treatment and maintained response. High expression of tumor PDL-1 was associated with improved responses (77). These studies showed that the phenomenon of hyper-progression may not be directly associated with PD-1/PDL-1 blockade in T cell lymphomas. Intralesional injection of TTI-621, a macrophage checkpoint inhibitor (anti CD47 antibody), in patients with CTCL showed 34% responses (78). Systemic administration study of this agent is underway (73).

## 4 Immune checkpoint blockade in multiple myeloma

Multiple myeloma (MM) is a plasma cell neoplasm accounting for approximately 10% of all hematological malignancies. Significant advances in the field include development and implementation of immunomodulatory drugs (IMIDs), proteosome inhibitors (PIs), anti-CD38 monoclonal antibodies and anti-BCMA therapies including chimeric receptor antigen T cells (CAR-T) and T cell engaging bispecific antibodies (BsAb). However, despite these significant advances, patients ultimately develop a progressively resistant disease. Immune checkpoint blockade was evaluated in this setting supported by pre-clinical studies which showed overexpression of PDL-1 in myeloma cells and CTLA4+ regulatory T cells in the tumor microenvironment.PDL-1 expression has also been shown to increase the risk of progression from monoclonal gammopathy to multiple myeloma (79–81). Despite the strong pre-clinical rationale, most studies of immune checkpoint blockade in myeloma have been disappointing.

Pembrolizumab was studied in intermediate/high risk smoldering multiple myeloma (SMM). Of the 13 patients treated, only one patient achieved a stringent CR with measurable residual disease (MRD) negativity while majority (85%) had stable disease. 3 patients discontinued treatment due to IRAEs (82). In a phase 1 study of pembrolizumab monotherapy for R/R MM, none of the 30 patients treated achieved an objective response. The best response achieved was stable disease with median duration of stable disease being 3.7 months (83). Nivolumab monotherapy in R/R MM patients lead to 4% objective response with majority (63%) achieving only stable disease (84). Safety profile of these agents in myeloma was congruent with other cancers. After failure of PD-1 blockade monotherapy to demonstrate efficacy, combinatorial approaches were also evaluated. Combination of nivolumab with ipilimumab in a small subset of RRMM patients did not show any objective responses (85). Lenalidomide was shown to enhance efficacy of PD-1/PDL-1 blockade by inhibition of myeloid derived suppressor cells (MDSC) mediated immune suppression in addition to direct downregulation of PD-1 expression on T and NK cells providing a framework for clinical evaluation of this combination (86, 87). Keynote-023 was a phase 1 study which showed that combination of pembrolizumab and lenalidomide and dexamethasone had ORR of 76% in heavily pre-treated R/R MM patients including lenalidomide refractory cases (88). Pembrolizumab in combination with pomalidomide and dexamethasone had an ORR of 60% with median duration of response being 14.7 months (89). Keynote-183 was a phase 3 RCT evaluating pomalidomide and dexamethasone with or without pembrolizumab in a predominantly lenalidomide refractory population (86%). This study was stopped given the risk of triple combination outweighed the benefit. Median PFS was 5.6 months with the triple combination as compared to 8.4 months in the other arm and there were increased serious treatment related adverse events (69% vs 46%) with pembrolizumab. 3% treatment related deaths were reported in the pembrolizumab arm (90). Keynote-185 looked at lenalidomide and dexamethasone with or without pembrolizumab in transplant ineligible newly diagnosed multiple myeloma (NDMM). Unfortunately, this study was also halted due to a higher mortality signal in the pembrolizumab arm attributed to myocarditis, large intestinal perforation, pneumonia, pulmonary embolism etc. Serious adverse events were increased in the pembrolizumab arm as well (54% vs 39%) (91). Nivolumab in combination with pomalidomide and dexamethasone was planned to be studied in RR MM however, it was discontinued after interim analysis showed HR > 1 for PFS and OS in the nivolumab arm (92). Combination of PD-1/PDL-1 monoclonal antibodies with other active myeloma drugs including carfilzomib (proteasome inhibitor), daratumumab (anti CD38 mab) and elotuzumab (anti SLAMF7 mab) are also being tested. Atezolizumab in combination with daratumumab and lenalidomide/pomalidomide showed very good partial response or better in 50% patients (3/6) (93). Early results from nivolumab-daratumumab study showed an ORR of 50% for the combination in RRMM (94). PD-1 blockade has also been shown to enhance ex-vivo T cell response to autologous dendritic cell/myeloma fusion vaccine and is being studied clinically in clinical context (95). The biological rationale for the increased adverse effects and mortality with PD-1 axis inhibitors is not completely understood. Unfortunately, this has led to discontinuation of several clinical trials of PD-1/PDL-1 inhibitors in MM. Some of the major challenges of PD-1/PDL-1 inhibitors in MM relate to dysfunctional T cell phenotype having decreased effector capacity and immunosuppressive micro-environment created by myeloid derived suppressor cells (MDSCs) and regulatory T cells. Despite the disappointing efficacy, patients achieving a response had a prolonged duration of response. Understanding the predictors of such responses would be key in optimizing patient selection for immune checkpoint therapy (96, 97). Please see **Table 3** for key trials in multiple myeloma.

**Table 3:** Key trials with Immune checkpoint inhibitors in plasma cell neoplasms.

Trial	Phase	Intervention	Checkpoint target	Population	Efficacy	Adverse events
CHECKMATE-039 (NCT01592370)	I	Nivolumab with ipilimumab	PD-1 and CTLA-4	R/R MM (n=5)	ORR 0%	29% grade 3-4 adverse events
KEYNOTE-013 (NCT01953692)	I	Pembrolizumab	PD-1	R/R MM (n=30)	ORR 0%	Myalgia (3%)
KEYNOTE-183 (NCT02576977)	III	Pembrolizumab with pomalidomide, dexamethasone vs pomalidomide, dexamethasone	PD-1	R/R MM (n=249)	mPFS 5.6 months vs 8.4 months	Serious adverse events 63% vs 46% Treatment related death 3% vs 0%
KEYNOTE-185	III	Pembrolizumab with lenalidomide, dexamethasone vs lenalidomide, dexamethasone	PD-1	NDMM (n=301)	6 month PFS 82% vs 85%	Serious adverse events 54% vs 39% Treatment related deaths 4% vs 1%

LAG-3 has been shown to be an important immune checkpoint in the pathogenesis and progression of myeloma. PD-1/LAG-3+ T cells are significantly enriched in patients with RRMM compared to NDMM (98, 99). Targeting the LAG-3/GAL-3 pathway has been shown to result in proliferation of MM specific T cells in combination with immunotherapy (100).

TIGIT has also been implicated in loss of myeloma specific immunity leading to disease progression. TIGIT blockade in combination with immunomodulators has been proposed as an exciting treatment modality to restore myeloma immunity (100). Exciting data from the MyCheckpoint study was recently presented at the AACR annual meeting. Anti TIGIT mab (BMS-986207) and anti LAG-3 mab (BMS-980616) were demonstrated to be safe alone and in combination with pomalidomide/dexamethasone. Objective response was noted in 2/6 patients treated with anti LAG-3 mab and in 3/6 patients treated with anti TIGIT mab (101). Longer follow up data is eagerly awaited.

TIM-3 is another checkpoint inhibitor proposed to play a role in T cell dysfunction and myeloma progression (102). TIM-3 pathway inhibition with anti-ligand antibodies led significantly higher NK cell cytolytic activity against myeloma cells and led to improved survival in a myeloma animal model (103). Clinical evaluation of TIM-3 inhibition in myeloma is warranted.

Myeloma cells have been shown to overexpress CD47 in a significantly larger amount compared to MGUS and SMM indicating a potential therapeutic target. Preclinical data has reported that CD47 blockade leads to induction of phagocytosis and killing of MM cells (104). Another correlative study of RRMM patients from the CoMMpass trial showed higher CD47 expression along with low CD38 expression correlated with worse overall survival and that dual inhibition of CD38 and CD47 had anti-tumor efficacy over single target inhibition (105). Clinical trials with CD47 inhibitors in myeloma area awaited.

## 5 Immune checkpoint blockade in myeloid malignancies

Immunotherapy in the form of allogeneic hematopoietic stem cell transplant (allo-HSCT) has been a pillar in the management of myeloid malignancies. For the treatment of most types of acute myeloid leukemia (AML) and myelodysplastic syndrome (MDS), allo-HSCT is the only known curative option. Because of the remarkable success of allo-HSCT in myeloid malignancies, there has been great interest in evaluating the therapeutic potential of other immunotherapies such as checkpoint inhibitors. Several trials have evaluated the efficacy of targeting pathways involving CTLA-4, PD-1, TIM-3 and CD47 in both AML and MDS.

Initial studies explored the use of ipilimumab in relapsed/refractory (r/r) AML and MDS in context of relapse post allo-HSCT (106–109). In a phase I/Ib trial, responses were observed in 5 out of 12 patients with relapsed AML after allo-HCT (108). Interestingly, of the 5 patients achieving CR, 4 had extramedullary disease and 3 responses lasted more than one year. Treatment was well tolerated with no major irAEs and no evidence of >grade 3 GVHD. Responses were related to bone marrow infiltration of cytotoxic CD8+ T cells and decreased systemic Treg activation (108, 109). In another phase 1b trial, non-transplanted patients with MDS who had failed hypomethylating agent therapy were treated with single-agent ipilimumab (110). Clinical activity of ipilimumab in this cohort of patients was very limited with only 1 out of the 29 patients achieving a CR. Of note, patients in this study only tolerated ipilimumab at a dose of 3mg/kg in contrast to the patients in the post-transplant trial (108) who tolerated a dose of 10mg/kg. This difference in dosing could account for the lack of response in the MDS trial. Most recently, a trial evaluating the combination of ipilimumab with decitabine in patients with AML and MDS (before and after allo-HSCT) reported encouraging results (111). The trial included 54 patients and demonstrated an overall response of 52% in patients who had not undergone transplant and 20% in patients post allo-HSCT. Similar to previous trials, most responses occurred at an ipilimumab dose of 10mg/kg. Despite meaningful response rates in this population, responses were short-lived with a median duration of response of 4.46 months and 6.14 in transplant-naïve and post-transplant patients respectively. From a safety standpoint, 11/25 patients in the post-transplant cohort had irAEs with 2 severe GVHD cases. In the transplant-naïve cohort, 11/23 patients reported irAEs. From a mechanistic standpoint, a parallel study concluded that decitabine acts on leukemic cells while ipilimimab acts on infiltrating lymphocytes and responses are determined by tumor cell burden and by the frequency and phenotype of infiltrating lymphocytes (111, 112).

Several studies have explored the use of pembrolizumab or nivolumab in myeloid malignancies. Given the known immune effects of hypomethylating agents (HMAs), most of these trials explored the efficacy of combination therapy using HMAs with pembrolizumab or nivolumab.

In a multi-center phase 2 trial, pembrolizumab was combined with azacytidine in patients with newly diagnosed and R/R AML (113). For the entire cohort (29 evaluable patients out of 37) the objective response rate (ORR) was found to be 55%. Significantly, for unfit patients with newly-diagnosed AML, the ORR was 94% with 47% of patients achieving a CR/CRi. In another study, patients with r/r AML were treated with pembrolizumab and decitabine (114). Although the number of patients was smaller (10 patients), the ORR was similar to the pembrolizumab/azacytidine study (113) at 60%. In general, initial studies indicate that the combination of pembrolizumab with HMAs is a safe option with meaningful clinical responses in patients with AML.

In another phase 2 study, Zeidner et al. evaluated the combination of pembrolizumab with high-dose cytarabine (HiDAC) (115). The hypothesis was that pembrolizumab would augment anti-leukemia T-cell responses and work synergistically with HiDAC. The study enrolled 37 patients and the ORR was 46% with a CR/CRi rate of 38%. Significantly, 50% of CR/CRi patients achieved MRD negativity. Grade ≥ 3 irAEs were not significant. Of note, the authors concluded that this combination of agents was most effective in patients who received this treatment as their first salvage regimen (106, 115).

The KEYNOTE-013 study assessed the single-agent activity of pembrolizumab in patients with MDS after HMA failure (116). Unfortunately, this study did not meet its primary endpoint since none of the patients achieved a CR or PR. Another phase 2 study evaluated the combination of azacytidine and pembrolizumab in MDS patients (117). This study included MDS patients that were either HMA-naïve or HMA failures. The ORR was 76% (CR rate of 18%) in HMA-naïve patients and 25% (CR rate of 5%) in HMA-failure patients. Consistent with previous data, pembrolizumab does not have meaningful clinical activity in MDS patients who have failed HMA therapy, but it is a promising option in patient who are HMA-naïve.

A phase 2 study evaluated the combination of nivolumab with azacytidine (118). The trial enrolled 70 patients and 45 had prior exposure to HMAs. The ORR was 33% with only 4 patients achieving a CR. irAEs were similar to other studies of CPIs in AML patients with 11% of patients reporting incidence of irAEs. Interestingly, correlative studies determined that CTLA-4 was upregulated in non-responders. As a result, another cohort was included in this trial which enrolled 31 AML patients treated with the triplet of nivolumab, ipilimumab and azacitidine (119). The ORR was 46% with a CR/CRi rate of 36%. The overall survival (OS) was 10.5 months compared to 4.6 months in the nivolumab/azacitidine cohort. Not surprisingly, the incidence of irAEs was increased in the triplet cohort with 25% of patients reporting irAEs.

Nivolumab has also been evaluated in the front-line setting in a phase 2 trial combining it with cytarabine and idarubicin in patients with newly-diagnosed AML and high-risk MDS (120). The CR rate was 78% with 79% of those patients achieving MRD negativity. Additionally, 19 patients were taken to allo-HCT. Grade 3-4 GVHD was noted in 5 patients. Interestingly, the OS was not significantly different between responders who were bridged to allo-HSCT and those that continued therapy with nivolumab. Authors postulated that this could indicate that the nivolumab was the potential ability to restore anti-leukemic immune surveillance and eradicate MRD. Immune dysregulation in myeloid malignancies is complex and a poorly understood subject (121). Blockade of PD-1 axis alone has poor efficacy which improves modestly in combination with hypomethylating agents or chemotherapy (122). Decreased MHC expression and negative regulation of T cells by way of increased alternative checkpoint expression, low T cell to MDS/AML cell ratio is thought to contribute to resistance to treatment, therefore effective therapies for tumor debulking and combination checkpoint blockade need further evaluation (112).

TIM-3 has been found to be upregulated on the surface of leukemia stem cells (LSCs) and leukemic blasts (123, 124). In addition, its expression is upregulated in malignant cells and it has been corelated with a poor clinical prognosis (125). All these findings make TIM-3 an attractive target in AML. Sabatolimab is a monoclonal anti-TIM-3 monoclonal antibody that is currently under investigation for the treatment of AML and MDS. Initial studies evaluated the combination of sabatolimab with HMAs in unfit patients with high-risk MDS and newly diagnosed AML (126, 127). In the MDS cohort, ORR was 57% with a CR rate of 43%. In the AML cohort, ORR was 40% with a CR/CRi rate of 30%. Responses were more durable in the MDS cohort with a median DOR of 16.1 months and 1-year PFS of 51.9% compared to a median DOR of 12.6 months and a 1-year PFS of 27.9% in the AML cohort. Meaningfully, subgroup analysis in this trial revealed that the response rates were preserved in patient with adverse risk mutations such as TP53, RUNX1 and ASXL1. In the AML cohort, in patients with at least one adverse risk mutation, the ORR was 53.8% with a median DOR of 12.6 months. In the MDS cohort, the ORR in patients with a TP53 mutation was 71.4% with a median DOR of 21.5 (127). Based on these data, there is a clear potential therapeutic effect in the use of sabatolimab in the treatment of AML and MDS and as a result the STIMULUS clinical trial program has been developed to study sabatolimab-based regimens in AML and MDS. Results from these studies will hopefully provide significant evidence showing a meaningful clinical effect of TIM-3 inhibition in AML and MDS.

In AML and MDS cells, it has been shown that CD47 is upregulated, and its upregulation is correlated with a poor clinical prognosis (128). Magrolimab is a humanized anti-CD47 monoclonal antibody that has been shown to induce macrophage-mediated phagocytosis of AML cells in preclinical studies (129). In clinical studies, it has been investigated as a single agent and in combination with HMAs for the treatment of AML. As a single agent, the responses were modest at best. In a phase I trial of single agent magrolimab in 15 r/r AML patients no CRs were achieved (130). Trials of the combination of magrolimab with azacytidine in AML and MDS showed more promising results (131, 132). In newly diagnosed, unfit AML patients, the ORR was 65% with a CR/CRi rate of 56%. In AML patients with a TP53 mutation, the ORR rate was 71% with a CR/CRi rate of 67%. In patients with untreated intermediate to very high risk MDS the ORR was 91% with a CR rate of 42%. These initial observations generated significant excitement in the field and several subsequent trials evaluating magrolimab in combinations with HMAs and/or venetoclax in patients with AML and MDS were initiated. Unfortunately, as of this writing the programs evaluating magrolimab in MDS have been halted due to futility and the programs evaluating magrolimab in AML are under a clinical hold by the FDA. Please see **Table 4** for key trials in myeloid neoplasms.

**Table 4:** Key trials with immune checkpoint inhibitors in myeloid neoplasms.

Trial	Phase	Intervention	Checkpoint target	Population	Efficacy	Adverse events (grade3-4)
NCT01822509	I	Ipilimumab	CTLA-4	Post AlloHCT relapse AML (n=12) MDS (n=2)	ORR 42%	IrAE 21% 14% GVHD
NCT02768792	I	Pembrolizumab	PD-1	R/R AML (n=37)	ORR 46% CRc 38% mOS 11.1 months	IrAE 14%
NCT03094637	II	Pembrolizumab	PD-1	HR MDS HMA naïve n=17 (Cohort A) HMA exposed n=20 (Cohort B)	Cohort A: ORR 76%, CR 18%. mOS NR Cohort B: ORR 25%, CR 5%. mOS 5.8 months	IrAE 43%
NCT02397720	II	Azacytidine with nivolumab with or without Ipilimumab	PD-1/CTLA-4	R/R AML Cohort 1: Aza+Nivo (n=70) Cohort 2: Aza+Nivo+ipi (n=31)	Cohort 1: ORR 33% Cohort 2: ORR 44% 1 year OS 45%	IrAE 25%
NCT02464657	II	Nivolumab with idarubicin+cytarabine	PD-1	Newly diagnosed AML/HR MDS (n=44)	ORR 77% mEFS not reached mOS 18.5 months	IrAE 14%
STIMULUS NCT03066648	I	Sabatolimab+HMA	TIM-3	Newly diagnosed HR MDS (n=53), AML (n=48)	MDS: ORR 57% mDOR 16 months AML: ORR 40% mDOR 12.6 months	IrAE 10%
ENHANCE NCT04313881	I	Magrolimab+HMA	CD47	R/R HR MDS (n=95)	ORR 75% CR 33% mDOR 9.8 months mOS NR	

Another anti-CD47 antibody, evorpacept, is currently under development. Evorpacept is reported to have significantly lower rate of on-target, off-tumor toxicity when compared to magrolimab as it has an inactive Fc domain (133). Evorpacept is being investigated in MDS and AML in the ASPEN clinical trial program. Results from these trials are eagerly awaited to provide further clarity regarding the therapeutic potential of CD47 blockade in AML and MDS.

## 6 Immune checkpoint blockade in the context of allogeneic HSCT

An important question in the field is whether the use of immune checkpoint inhibitors before or after an allogeneic transplantation improves outcomes, or whether this would result in an increase in toxicity due to GVHD. In initial studies, it was reported that Hodgkin lymphoma patients treated with an immune checkpoint inhibitor who subsequently underwent and allo-HSCT had an increased risk of complications including hyper-acute GVHD (134–136). Most recently, several studies have evaluated the use of immune checkpoint inhibitors before and after allo-HSCT in AML and MDS patients. Significantly, these studies have found no evidence of increased severe immune-related toxicities in AML/MDS patients treated with immune checkpoint inhibitors before or after allo-HSCT especially with the use of post-transplant cyclophosphamide as GVHD prophylaxis (120, 126, 137–140).

## 7 Toxicities of immune checkpoint blockade

Immune related adverse event (IrAE) are a unique group of toxicities associated with immune checkpoint inhibitors. Different from toxicities of conventional cytotoxic chemotherapy, pathophysiology of these IrAEs is not completely understood. Given the mechanism of action of checkpoint inhibitors, IrAEs are thought to be mediated by increased T cell over activation and cytokine release (141, 142). These can be systemic adverse events presenting as fatigue or in severe forms cytokine release syndrome but more commonly are organ limited toxicities. IrAEs commonly involve the skin, GI tract, thyroid, liver, and the lungs but can essentially involve any organ. The majority of the IrAEs noted in clinical trials for hematological malignancies were mild to moderate and were able to be managed symptomatically or with corticosteroids. However, there are severe cases of IrAEs presenting as pneumonitis, myocarditis, encephalitis, nephritis, Steven-Johnson syndrome etc. reported which led to death or significant comorbidity. Most of the data for IrAEs specifically in hematological malignancies come from the early phase clinical trials as discussed previously. The two phase 3 studies including PD-1 inhibitor pembrolizumab in combination with immunomodulatory drugs in patients with multiple myeloma were Keynote-183 and Keynote-185. Both these studies were halted due to increased treatment related deaths in the pembrolizumab+IMID arms related to severe IrAEs (myocarditis, pneumonitis, Steven Johnson Syndrome etc) (90, 91). Whether the toxicity was exacerbated in combination with an IMID is a debatable question. In case of cHL, in the SWOG 1826 trial comparing Nivolumab-AVD to BV-AVD, Nivolumab-AVD was tolerated well and had a better safety profile compared to the control arm apart from hypo/hyperthyroidism which was manageable. There are multiple reviews discussing mechanisms and managements of checkpoint inhibition associated IrAEs in detail (142–144).

CD47 is critical to red blood cell homeostasis and targeting CD47 can potentially cause an ‘on-target off-tumor’ adverse effect of anemia. Therefore, an initial ‘priming’ dose of magrolimab (anti-CD47 mAb) has been proposed to assess anemia before proceeding to higher doses (145). Regardless of this strategy the most common magrolimab related adverse event is anemia (50-70%) (72, 145, 146).

## 8 Conclusion

Harnessing the potency of the immune system to target hematological malignancies has resulted in dramatic improvement in outcomes for patients with hematological malignancies. The discovery of negative checkpoint pathways and their role in muting immune mediating targeting of tumor has been instrumental, and the development of immune checkpoint inhibitors has resulted in major therapeutic advances. While checkpoint blockers have shown efficacy in cHL and PMBCL, only limited efficacy was seen in non-Hodgkin lymphoma, plasma cell disorders and myeloid malignancies. There is significant room for improvement in development of immune checkpoint blockers in hematological malignancies. Efforts are underway to develop bispecific antibodies targeting two checkpoint molecules, checkpoint molecule and costimulatory molecule, checkpoint molecule and tumor target antigen etc. (147–151) Combination of checkpoint blockers with novel antibody drug conjugates are also a major area of research (152, 153). Most of these therapies are in the preclinical phase of development and data in the context of hematological malignancies is not available. Future directions will focus on understanding biomarkers predictive of response and resistance, on understanding the optimal timing for incorporating immune checkpoint blockade in the course of disease, and on developing novel combinatorial strategies.

## Author contributions

PP: Writing – original draft. JV: Writing – original draft, Writing – review & editing. JR: Writing – review & editing.

## References

[1] Weiden PL Flournoy N Thomas ED Prentice R Fefer A Buckner CD. Antileukemic effect of graft-versus-host disease in human recipients of allogeneic-marrow grafts. N Engl J Med (1979) 300(19):1068–73. doi: 10.1056/NEJM197905103001902 34792

[2] van den Broek T Borghans JAM van Wijk F. The full spectrum of human naive T cells. Nat Rev Immunol (2018) 18(6):363–73. doi: 10.1038/s41577-018-0001-y 29520044

[3] June CH Ledbetter JA Linsley PS Thompson CB. Role of the CD28 receptor in T-cell activation. Immunol Today (1990) 11(6):211–6. doi: 10.1016/0167-5699(90)90085-N 2162180

[4] Hansen JA Martin PJ Nowinski RC. Monoclonal antibodies identifying a novel T-Cell antigen and Ia antigens of human lymphocytes. Immunogenetics (1980) 10(1):247–60. doi: 10.1007/BF01561573

[5] Krummel MF Allison JP. CD28 and CTLA-4 have opposing effects on the response of T cells to stimulation. J Exp Med (1995) 182(2):459–65. doi: 10.1084/jem.182.2.459 PMC2192127 7543139

[6] Linsley PS Brady W Urnes M Grosmaire LS Damle NK Ledbetter JA. CTLA-4 is a second receptor for the B cell activation antigen B7. J Exp Med (1991) 174(3):561–9. doi: 10.1084/jem.174.3.561 PMC2118936 1714933

[7] Chuang E Lee KM Robbins MD Duerr JM Alegre ML Hambor JE. Regulation of cytotoxic T lymphocyte-associated molecule-4 by Src kinases. J Immunol (1999) 162(3):1270–7. doi: 10.4049/jimmunol.162.3.1270 9973379

[8] Leach DR Krummel MF Allison JP. Enhancement of antitumor immunity by CTLA-4 blockade. Science (1996) 271(5256):1734–6. doi: 10.1126/science.271.5256.1734 8596936

[9] Hodi FS O'Day SJ McDermott DF Weber RW Sosman JA Haanen JB. Improved survival with ipilimumab in patients with metastatic melanoma. N Engl J Med (2010) 363(8):711–23. doi: 10.1056/NEJMoa1003466 PMC3549297 20525992

[10] Ishida Y Agata Y Shibahara K Honjo T. Induced expression of PD-1, a novel member of the immunoglobulin gene superfamily, upon programmed cell death. EMBO J (1992) 11(11):3887–95. doi: 10.1002/j.1460-2075.1992.tb05481.x PMC556898 1396582

[11] Freeman GJ Long AJ Iwai Y Bourque K Chernova T Nishimura H. Engagement of the PD-1 immunoinhibitory receptor by a novel B7 family member leads to negative regulation of lymphocyte activation. J Exp Med (2000) 192(7):1027–34. doi: 10.1084/jem.192.7.1027 PMC2193311 11015443

[12] Hui E Cheung J Zhu J Su X Taylor MJ Wallweber HA. T cell costimulatory receptor CD28 is a primary target for PD-1-mediated inhibition. Science (2017) 355(6332):1428–33. doi: 10.1126/science.aaf1292 PMC6286077 28280247

[13] Barber DL Wherry EJ Masopust D Zhu B Allison JP Sharpe AH. Restoring function in exhausted CD8 T cells during chronic viral infection. Nature (2006) 439(7077):682–7. doi: 10.1038/nature04444 16382236

[14] Triebel F Jitsukawa S Baixeras E Roman-Roman S Genevee C Viegas-Pequignot E. LAG-3, a novel lymphocyte activation gene closely related to CD4. J Exp Med (1990) 171(5):1393–405. doi: 10.1084/jem.171.5.1393 PMC2187904 1692078

[15] Maruhashi T Okazaki IM Sugiura D Takahashi S Maeda TK Shimizu K. LAG-3 inhibits the activation of CD4(+) T cells that recognize stable pMHCII through its conformation-dependent recognition of pMHCII. Nat Immunol (2018) 19(12):1415–26. doi: 10.1038/s41590-018-0217-9 30349037

[16] Wang J Sanmamed MF Datar I Su TT Ji L Sun J. Fibrinogen-like protein 1 is a major immune inhibitory ligand of LAG-3. Cell (2019) 176(1-2):334–47.e12. doi: 10.1016/j.cell.2018.11.010 30580966 PMC6365968

[17] Maruhashi T Sugiura D Okazaki IM Shimizu K Maeda TK Ikubo J. Binding of LAG-3 to stable peptide-MHC class II limits T cell function and suppresses autoimmunity and anti-cancer immunity. Immunity (2022) 55(5):912–24.e8. doi: 10.1016/j.immuni.2022.03.013 35413245

[18] Huang YH Zhu C Kondo Y Anderson AC Gandhi A Russell A. CEACAM1 regulates TIM-3-mediated tolerance and exhaustion. Nature (2015) 517(7534):386–90. doi: 10.1038/nature13848 PMC4297519 25363763

[19] Zeidan AM Komrokji RS Brunner AM. TIM-3 pathway dysregulation and targeting in cancer. Expert Rev Anticancer Ther (2021) 21(5):523–34. doi: 10.1080/14737140.2021.1865814 33334180

[20] Sakuishi K Apetoh L Sullivan JM Blazar BR Kuchroo VK Anderson AC. Targeting Tim-3 and PD-1 pathways to reverse T cell exhaustion and restore anti-tumor immunity. J Exp Med (2010) 207(10):2187–94. doi: 10.1084/jem.20100643 PMC2947065 20819927

[21] Koyama S Akbay EA Li YY Herter-Sprie GS Buczkowski KA Richards WG. Adaptive resistance to therapeutic PD-1 blockade is associated with upregulation of alternative immune checkpoints. Nat Commun (2016) 7:10501. doi: 10.1038/ncomms10501 26883990 PMC4757784

[22] Yu X Harden K Gonzalez LC Francesco M Chiang E Irving B. The surface protein TIGIT suppresses T cell activation by promoting the generation of mature immunoregulatory dendritic cells. Nat Immunol (2009) 10(1):48–57. doi: 10.1038/ni.1674 19011627

[23] Levin SD Taft DW Brandt CS Bucher C Howard ED Chadwick EM. Vstm3 is a member of the CD28 family and an important modulator of T-cell function. Eur J Immunol (2011) 41(4):902–15. doi: 10.1002/eji.201041136 PMC3733993 21416464

[24] Jin S Zhang Y Zhou F Chen X Sheng J Zhang J. TIGIT: A promising target to overcome the barrier of immunotherapy in hematological Malignancies. Front Oncol (2022) 12. doi: 10.3389/fonc.2022.1091782 PMC9807865 36605439

[25] Cai L Li Y Tan J Xu L Li Y. Targeting LAG-3, TIM-3, and TIGIT for cancer immunotherapy. J Hematol Oncol (2023) 16(1):101. doi: 10.1186/s13045-023-01499-1 37670328 PMC10478462

[26] Ferragut F Alcaraz PB Beati P Girard MC Ossowski MS Chadi R. Expression of Inhibitory Receptors TIGIT, TIM-3, and LAG-3 on CD4+ T cells from patients with different clinical forms of chronic chagas disease. J Immunol (2023) 210(5):568–79. doi: 10.4049/jimmunol.2200436 36602929

[27] Pittet MJ Michielin O Migliorini D. Clinical relevance of tumour-associated macrophages. Nat Rev Clin Oncol (2022) 19(6):402–21. doi: 10.1038/s41571-022-00620-6 35354979

[28] Korolnek T Hamza I. Macrophages and iron trafficking at the birth and death of red cells. Blood (2015) 125(19):2893–7. doi: 10.1182/blood-2014-12-567776 PMC4424413 25778532

[29] Jaiswal S Jamieson CH Pang WW Park CY Chao MP Majeti R. CD47 is upregulated on circulating hematopoietic stem cells and leukemia cells to avoid phagocytosis. Cell (2009) 138(2):271–85. doi: 10.1016/j.cell.2009.05.046 PMC2775564 19632178

[30] Liu X Pu Y Cron K Deng L Kline J Frazier WA. CD47 blockade triggers T cell-mediated destruction of immunogenic tumors. Nat Med (2015) 21(10):1209–15. doi: 10.1038/nm.3931 PMC4598283 26322579

[31] Vonderheide RH. CD47 blockade as another immune checkpoint therapy for cancer. Nat Med (2015) 21(10):1122–3. doi: 10.1038/nm.3965 26444633

[32] Chen BJ Chapuy B Ouyang J Sun HH Roemer MG Xu ML. PD-L1 expression is characteristic of a subset of aggressive B-cell lymphomas and virus-associated Malignancies. Clin Cancer Res (2013) 19(13):3462–73. doi: 10.1158/1078-0432.CCR-13-0855 PMC4102335 23674495

[33] Ansell SM Radford J Connors JM Długosz-Danecka M Kim WS Gallamini A. Overall survival with brentuximab vedotin in stage III or IV hodgkin's lymphoma. N Engl J Med (2022) 387(4):310–20. doi: 10.1056/NEJMoa2206125 35830649

[34] Armand P Engert A Younes A Fanale M Santoro A Zinzani PL. Nivolumab for relapsed/refractory classic hodgkin lymphoma after failure of autologous hematopoietic cell transplantation: extended follow-up of the multicohort single-arm phase II checkMate 205 trial. J Clin Oncol (2018) 36(14):1428–39. doi: 10.1200/JCO.2017.76.0793 PMC6075855 29584546

[35] Armand P Zinzani PL Lee HJ Johnson NA Brice P Radford J. Five-year follow-up of KEYNOTE-087: pembrolizumab monotherapy in relapsed/refractory classical Hodgkin lymphoma. Blood (2023) 142(10):878–86. doi: 10.1182/blood.2022019386 PMC10624931 37319435

[36] Kuruvilla J Ramchandren R Santoro A Paszkiewicz-Kozik E Gasiorowski R Johnson NA. Pembrolizumab versus brentuximab vedotin in relapsed or refractory classical Hodgkin lymphoma (KEYNOTE-204): an interim analysis of a multicentre, randomised, open-label, phase 3 study. Lancet Oncol (2021) 22(4):512–24. doi: 10.1016/S1470-2045(21)00005-X 33721562

[37] Moskowitz AJ Shah G Schöder H Ganesan N Drill E Hancock H. Phase II trial of pembrolizumab plus gemcitabine, vinorelbine, and liposomal doxorubicin as second-line therapy for relapsed or refractory classical hodgkin lymphoma. J Clin Oncol (2021) 39(28):3109–17. doi: 10.1200/JCO.21.01056 PMC9851707 34170745

[38] Mei M Palmer J Tsai N-C Lee HJ Isufi I Popplewell LL. Nivolumab plus ICE as first salvage therapy in high-risk relapsed/refractory hodgkin lymphoma. Blood (2022) 140(Supplement 1):774–6. doi: 10.1182/blood-2022-167626

[39] Armand P Chen YB Redd RA Joyce RM Bsat J Jeter E. PD-1 blockade with pembrolizumab for classical Hodgkin lymphoma after autologous stem cell transplantation. Blood (2019) 134(1):22–9. doi: 10.1182/blood.2019000215 PMC6609955 30952672

[40] Bachier C SChade H Zoghi B Ramakrishnan A Shah NN. A Phase II Single Arm Study of Nivolumab As Maintenance Therapy after Autologous Stem Cell Transplantation in Patients with Hodgkin Lymphoma at Risk of Relapse or Progression. Blood (2021) 138:2455. doi: 10.1182/blood-2021-148139 33945606

[41] Herrera AF Burton C Radford J Miall F Townsend W Santoro A. Avelumab in relapsed/refractory classical Hodgkin lymphoma: phase 1b results from the JAVELIN Hodgkins trial. Blood Adv (2021) 5(17):3387–96. doi: 10.1182/bloodadvances.2021004511 PMC8525219 34477818

[42] Advani RH Moskowitz AJ Bartlett NL Vose JM Ramchandren R Feldman TA. Brentuximab vedotin in combination with nivolumab in relapsed or refractory Hodgkin lymphoma: 3-year study results. Blood (2021) 138(6):427–38. doi: 10.1182/blood.2020009178 PMC12684812 33827139

[43] Herrera AF LeBlanc ML Castellino SM Li H Rutherford SC Evens AM. SWOG S1826, a randomized study of nivolumab(N)-AVD versus brentuximab vedotin(BV)-AVD in advanced stage (AS) classic Hodgkin lymphoma (HL). J Clin Oncol (2023) 41(17_suppl):LBA4–LBA. doi: 10.1200/JCO.2023.41.17_suppl.LBA4

[44] Diefenbach CS Hong F Ambinder RF Cohen JB Robertson MJ David KA. Ipilimumab, nivolumab, and brentuximab vedotin combination therapies in patients with relapsed or refractory Hodgkin lymphoma: phase 1 results of an open-label, multicentre, phase 1/2 trial. Lancet Haematol (2020) 7(9):e660–e70. doi: 10.1016/S2352-3026(20)30221-0 PMC7737486 32853585

[45] Johnson NA Lavie D Borchmann P Gregory GP Herrera AF Minuk L. Updated results from an open-label phase 1/2 study of favezelimab (anti-LAG-3) plus pembrolizumab in relapsed or refractory classical hodgkin lymphoma. Blood (2022) 140(Supplement 1):6540–2. doi: 10.1182/blood-2022-166846

[46] Yang ZZ Novak AJ Stenson MJ Witzig TE Ansell SM. Intratumoral CD4+CD25+ regulatory T-cell-mediated suppression of infiltrating CD4+ T cells in B-cell non-Hodgkin lymphoma. Blood (2006) 107(9):3639–46. doi: 10.1182/blood-2005-08-3376 PMC1895773 16403912

[47] Ansell SM Hurvitz SA Koenig PA LaPlant BR Kabat BF Fernando D. Phase I study of ipilimumab, an anti–CTLA-4 monoclonal antibody, in patients with relapsed and refractory B-cell non–hodgkin lymphoma. Clin Cancer Res (2009) 15(20):6446–53. doi: 10.1158/1078-0432.CCR-09-1339 PMC2763019 19808874

[48] Tuscano JM Maverakis E Groshen S Tsao-Wei D Luxardi G Merleev AA. A phase I study of the combination of rituximab and ipilimumab in patients with relapsed/refractory B-cell lymphoma. Clin Cancer Res (2019) 25(23):7004–13. doi: 10.1158/1078-0432.CCR-19-0438 PMC7354236 31481504

[49] Armand P Lesokhin A Borrello I Timmerman J Gutierrez M Zhu L. A phase 1b study of dual PD-1 and CTLA-4 or KIR blockade in patients with relapsed/refractory lymphoid Malignancies. Leukemia (2021) 35(3):777–86. doi: 10.1038/s41375-020-0939-1 PMC7932914 32601377

[50] Godfrey J Tumuluru S Bao R Leukam M Venkataraman G Phillip J. PD-L1 gene alterations identify a subset of diffuse large B-cell lymphoma harboring a T-cell-inflamed phenotype. Blood (2019) 133(21):2279–90. doi: 10.1182/blood-2018-10-879015 PMC6911840 30910787

[51] Kiyasu J Miyoshi H Hirata A Arakawa F Ichikawa A Niino D. Expression of programmed cell death ligand 1 is associated with poor overall survival in patients with diffuse large B-cell lymphoma. Blood (2015) 126(19):2193–201. doi: 10.1182/blood-2015-02-629600 PMC4635115 26239088

[52] Ansell SM Minnema MC Johnson P Timmerman JM Armand P Shipp MA. Nivolumab for relapsed/refractory diffuse large B-cell lymphoma in patients ineligible for or having failed autologous transplantation: A single-arm, phase II study. J Clin Oncol (2019) 37(6):481–9. doi: 10.1200/JCO.18.00766 PMC6528729 30620669

[53] Armand P Janssens A Gritti G Radford J Timmerman J Pinto A. Efficacy and safety results from CheckMate 140, a phase 2 study of nivolumab for relapsed/refractory follicular lymphoma. Blood (2021) 137(5):637–45. doi: 10.1182/blood.2019004753 PMC7869188 32870269

[54] Nastoupil LJ Chin CK Westin JR Fowler NH Samaniego F Cheng X. Safety and activity of pembrolizumab in combination with rituximab in relapsed or refractory follicular lymphoma. Blood Adv (2022) 6(4):1143–51. doi: 10.1182/bloodadvances.2021006240 PMC8864656 35015819

[55] Morschhauser F Ghosh N Lossos IS Palomba ML Mehta A Casasnovas O. Obinutuzumab-atezolizumab-lenalidomide for the treatment of patients with relapsed/refractory follicular lymphoma: final analysis of a Phase Ib/II trial. Blood Cancer J (2021) 11(8):147. doi: 10.1038/s41408-021-00539-8 34417444 PMC8379261

[56] Herrera AF Goy A Mehta A Ramchandren R Pagel JM Svoboda J. Safety and activity of ibrutinib in combination with durvalumab in patients with relapsed or refractory follicular lymphoma or diffuse large B-cell lymphoma. Am J Hematol (2020) 95(1):18–27. doi: 10.1002/ajh.25659 31621094 PMC6904508

[57] Nowakowski GS Willenbacher W Greil R Larsen TS Patel K Jäger U. Safety and efficacy of durvalumab with R-CHOP or R2-CHOP in untreated, high-risk DLBCL: a phase 2, open-label trial. Int J Hematol (2022) 115(2):222–32. doi: 10.1007/s12185-021-03241-4 34797531

[58] Ding W LaPlant BR Call TG Parikh SA Leis JF He R. Pembrolizumab in patients with CLL and Richter transformation or with relapsed CLL. Blood (2017) 129(26):3419–27. doi: 10.1182/blood-2017-02-765685 PMC5492091 28424162

[59] Gregory GP Kumar S Wang D Mahadevan D Walker P Wagner-Johnston N. Pembrolizumab plus dinaciclib in patients with hematologic Malignancies: the phase 1b KEYNOTE-155 study. Blood Adv (2022) 6(4):1232–42. doi: 10.1182/bloodadvances.2021005872 PMC8864641 34972202

[60] Frigault MJ Armand P Redd RA Jeter E Merryman RW Coleman KC. PD-1 blockade for diffuse large B-cell lymphoma after autologous stem cell transplantation. Blood Adv (2020) 4(1):122–6. doi: 10.1182/bloodadvances.2019000784 PMC6960482 31917843

[61] Chong EA Alanio C Svoboda J Nasta SD Landsburg DJ Lacey SF. Pembrolizumab for B-cell lymphomas relapsing after or refractory to CD19-directed CAR T-cell therapy. Blood (2022) 139(7):1026–38. doi: 10.1182/blood.2021012634 PMC9211527 34496014

[62] Zinzani PL Ribrag V Moskowitz CH Michot JM Kuruvilla J Balakumaran A. Safety and tolerability of pembrolizumab in patients with relapsed/refractory primary mediastinal large B-cell lymphoma. Blood (2017) 130(3):267–70. doi: 10.1182/blood-2016-12-758383 PMC5766837 28490569

[63] Zinzani PL Thieblemont C Melnichenko V Bouabdallah K Walewski J Majlis A. Pembrolizumab in relapsed or refractory primary mediastinal large B-cell lymphoma: final analysis of KEYNOTE-170. Blood (2023) 142(2):141–5. doi: 10.1182/blood.2022019340 PMC10651864 37130017

[64] Zinzani PL Santoro A Gritti G Brice P Barr PM Kuruvilla J. Nivolumab combined with brentuximab vedotin for relapsed/refractory primary mediastinal large B-cell lymphoma: efficacy and safety from the phase II checkMate 436 study. J Clin Oncol (2019) 37(33):3081–9. doi: 10.1200/JCO.19.01492 PMC6864847 31398081

[65] Salik B Smyth MJ Nakamura K. Targeting immune checkpoints in hematological Malignancies. J Hematol Oncol (2020) 13(1):111. doi: 10.1186/s13045-020-00947-6 32787882 PMC7425174

[66] Zhang C Wang L Xu C Xu H Wu Y. Resistance mechanisms of immune checkpoint inhibition in lymphoma: Focusing on the tumor microenvironment. Front Pharmacol (2023) 14. doi: 10.3389/fphar.2023.1079924 PMC10027765 36959853

[67] Yang ZZ Grote DM Ziesmer SC Niki T Hirashima M Novak AJ. IL-12 upregulates TIM-3 expression and induces T cell exhaustion in patients with follicular B cell non-Hodgkin lymphoma. J Clin Invest (2012) 122(4):1271–82. doi: 10.1172/JCI59806 PMC3314462 22426209

[68] James G Xiufen C Nicole S Alan C Jovian Y Arina V. TIGIT is a key inhibitory checkpoint receptor in lymphoma. J ImmunoTher Cancer (2023) 11(6):e006582. doi: 10.1136/jitc-2022-006582 37364933 PMC10410806

[69] Chao MP Tang C Pachynski RK Chin R Majeti R Weissman IL. Extranodal dissemination of non-Hodgkin lymphoma requires CD47 and is inhibited by anti-CD47 antibody therapy. Blood (2011) 118(18):4890–901. doi: 10.1182/blood-2011-02-338020 PMC3208297 21828138

[70] Chao MP Alizadeh AA Tang C Myklebust JH Varghese B Gill S. Anti-CD47 antibody synergizes with rituximab to promote phagocytosis and eradicate non-Hodgkin lymphoma. Cell (2010) 142(5):699–713. doi: 10.1016/j.cell.2010.07.044 20813259 PMC2943345

[71] Cao X Wang Y Zhang W Zhong X Gunes EG Dang J. Targeting macrophages for enhancing CD47 blockade–elicited lymphoma clearance and overcoming tumor-induced immunosuppression. Blood (2022) 139(22):3290–302. doi: 10.1182/blood.2021013901 PMC9164740 35134139

[72] Advani R Flinn I Popplewell L Forero A Bartlett NL Ghosh N. CD47 blockade by hu5F9-G4 and rituximab in non-hodgkin’s lymphoma. N Engl J Med (2018) 379(18):1711–21. doi: 10.1056/NEJMoa1807315 PMC8058634 30380386

[73] Ansell SM Maris MB Lesokhin AM Chen RW Flinn IW Sawas A. Phase I study of the CD47 blocker TTI-621 in patients with relapsed or refractory hematologic Malignancies. Clin Cancer Res (2021) 27(8):2190–9. doi: 10.1158/1078-0432.CCR-20-3706 33451977

[74] Patel K Maris MB Cheson BD Zonder JA Lesokhin AM Von Keudell G. Ongoing, first-in-human, phase I dose escalation study of the investigational CD47-blocker TTI-622 in patients with advanced relapsed or refractory lymphoma. J Clin Oncol (2020) 38(15_suppl):3030–. doi: 10.1200/JCO.2020.38.15_suppl.3030

[75] Bennani NN Kim HJ Pederson LD Atherton PJ Micallef IN Thanarajasingam G. Nivolumab in patients with relapsed or refractory peripheral T-cell lymphoma: modest activity and cases of hyperprogression. J Immunother Cancer (2022) 10(6). doi: 10.1136/jitc-2022-004984 PMC9234908 35750419

[76] Khodadoust MS Rook AH Porcu P Foss F Moskowitz AJ Shustov A. Pembrolizumab in relapsed and refractory mycosis fungoides and sézary syndrome: A multicenter phase II study. J Clin Oncol (2020) 38(1):20–8. doi: 10.1200/JCO.19.01056 PMC6943974 31532724

[77] Kim SJ Lim JQ Laurensia Y Cho J Yoon SE Lee JY. Avelumab for the treatment of relapsed or refractory extranodal NK/T-cell lymphoma: an open-label phase 2 study. Blood (2020) 136(24):2754–63. doi: 10.1182/blood.2020007247 32766875

[78] Querfeld C Thompson JA Taylor MH DeSimone JA Zain JM Shustov AR. Intralesional TTI-621, a novel biologic targeting the innate immune checkpoint CD47, in patients with relapsed or refractory mycosis fungoides or Sézary syndrome: a multicentre, phase 1 study. Lancet Haematol (2021) 8(11):e808–e17. doi: 10.1016/S2352-3026(21)00271-4 34627593

[79] Liu J Hamrouni A Wolowiec D Coiteux V Kuliczkowski K Hetuin D. Plasma cells from multiple myeloma patients express B7-H1 (PD-L1) and increase expression after stimulation with IFN-{gamma} and TLR ligands *via* a MyD88-, TRAF6-, and MEK-dependent pathway. Blood (2007) 110(1):296–304. doi: 10.1182/blood-2006-10-051482 17363736

[80] Braga WMT Vettore AL Carvalho AC Atanackovic D Colleoni GWB. Overexpression of CTLA-4 in the bone marrow of patients with multiple myeloma as a sign of local accumulation of immunosuppressive tregs – perspectives for novel treatment strategies. Blood (2011) 118(21):1829–. doi: 10.1182/blood.V118.21.1829.1829

[81] Dhodapkar MV Sexton R Das R Dhodapkar KM Zhang L Sundaram R. Prospective analysis of antigen-specific immunity, stem-cell antigens, and immune checkpoints in monoclonal gammopathy. Blood (2015) 126(22):2475–8. doi: 10.1182/blood-2015-03-632919 PMC4661169 26468228

[82] Manasanch EE Han G Mathur R Qing Y Zhang Z Lee H. A pilot study of pembrolizumab in smoldering myeloma: report of the clinical, immune, and genomic analysis. Blood Adv (2019) 3(15):2400–8. doi: 10.1182/bloodadvances.2019000300 PMC6693011 31405950

[83] Ribrag V Avigan DE Green DJ Wise-Draper T Posada JG Vij R. Phase 1b trial of pembrolizumab monotherapy for relapsed/refractory multiple myeloma: KEYNOTE-013. Br J Haematol (2019) 186(3):e41–e4. doi: 10.1111/bjh.15888 30937889

[84] Lesokhin AM Ansell SM Armand P Scott EC Halwani A Gutierrez M. Nivolumab in patients with relapsed or refractory hematologic Malignancy: preliminary results of a phase ib study. J Clin Oncol (2016) 34(23):2698–704. doi: 10.1200/JCO.2015.65.9789 PMC5019749 27269947

[85] Ansell S Gutierrez ME Shipp MA Gladstone D Moskowitz A Borello I. A phase 1 study of nivolumab in combination with ipilimumab for relapsed or refractory hematologic Malignancies (CheckMate 039). Blood (2016) 128(22):183–. doi: 10.1182/blood.V128.22.183.183

[86] Görgün G Samur MK Cowens KB Paula S Bianchi G Anderson JE. Lenalidomide enhances immune checkpoint blockade-induced immune response in multiple myeloma. Clin Cancer Res (2015) 21(20):4607–18. doi: 10.1158/1078-0432.CCR-15-0200 PMC4609232 25979485

[87] Benson DM Jr. Bakan CE Mishra A Hofmeister CC Efebera Y Becknell B. The PD-1/PD-L1 axis modulates the natural killer cell versus multiple myeloma effect: a therapeutic target for CT-011, a novel monoclonal anti-PD-1 antibody. Blood (2010) 116(13):2286–94. doi: 10.1182/blood-2010-02-271874 PMC3490105 20460501

[88] Mateos MV Orlowski RZ Ocio EM Rodríguez-Otero P Reece D Moreau P. Pembrolizumab combined with lenalidomide and low-dose dexamethasone for relapsed or refractory multiple myeloma: phase I KEYNOTE-023 study. Br J Haematol (2019) 186(5):e117–e21. doi: 10.1111/bjh.15946 31090915

[89] Badros A Hyjek E Ma N Lesokhin A Dogan A Rapoport AP. Pembrolizumab, pomalidomide, and low-dose dexamethasone for relapsed/refractory multiple myeloma. Blood (2017) 130(10):1189–97. doi: 10.1182/blood-2017-03-775122 28461396

[90] Mateos MV Blacklock H Schjesvold F Oriol A Simpson D George A. Pembrolizumab plus pomalidomide and dexamethasone for patients with relapsed or refractory multiple myeloma (KEYNOTE-183): a randomised, open-label, phase 3 trial. Lancet Haematol (2019) 6(9):e459–e69. doi: 10.1016/S2352-3026(19)30110-3 31327687

[91] Usmani SZ Schjesvold F Oriol A Karlin L Cavo M Rifkin RM. Pembrolizumab plus lenalidomide and dexamethasone for patients with treatment-naive multiple myeloma (KEYNOTE-185): a randomised, open-label, phase 3 trial. Lancet Haematol (2019) 6(9):e448–e58. doi: 10.1016/S2352-3026(19)30109-7 31327689

[92] Lesokhin AM Bal S Badros AZ. Lessons learned from checkpoint blockade targeting PD-1 in multiple myeloma. Cancer Immunol Res (2019) 7(8):1224–9. doi: 10.1158/2326-6066.CIR-19-0148 PMC6891823 31371317

[93] Cho HJ Costa LJ Davies FE Neparidze N Vij R Feng Y. Atezolizumab in combination with daratumumab with or without lenalidomide or pomalidomide: A phase ib study in patients with multiple myeloma. Blood (2018) 132(Supplement 1):597–. doi: 10.1182/blood-2018-99-114960

[94] Verkleij CPM Minnema MC de Weerdt O Bosman PWC Frerichs KA Croockewit AJ. Efficacy and safety of nivolumab combined with daratumumab with or without low-dose cyclophosphamide in relapsed/refractory multiple myeloma; interim analysis of the phase 2 nivo-dara study. Blood (2019) 134(Supplement_1):1879–. doi: 10.1182/blood-2019-124339

[95] Rosenblatt J Glotzbecker B Mills H Vasir B Tzachanis D Levine JD. PD-1 blockade by CT-011, anti-PD-1 antibody, enhances ex vivo T-cell responses to autologous dendritic cell/myeloma fusion vaccine. J Immunother (2011) 34(5):409–18. doi: 10.1097/CJI.0b013e31821ca6ce PMC3142955 21577144

[96] Chung DJ Pronschinske KB Shyer JA Sharma S Leung S Curran SA. T-cell exhaustion in multiple myeloma relapse after autotransplant: optimal timing of immunotherapy. Cancer Immunol Res (2016) 4(1):61–71. doi: 10.1158/2326-6066.CIR-15-0055 26464015 PMC4703436

[97] Russell BM Avigan DE. Immune dysregulation in multiple myeloma: the current and future role of cell-based immunotherapy. Int J Hematol (2023) 117(5):652–9. doi: 10.1007/s12185-023-03579-x PMC10039687 36964840

[98] Chen M Zhu J Yang X Yao J Liu Y Liu Q. PD-1 and LAG-3-positive T cells are associated with clinical outcomes of relapsed/refractory multiple myeloma patients. Eur J Med Res (2022) 27(1):296. doi: 10.1186/s40001-022-00923-5 36529769 PMC9761990

[99] Kreiniz N Eiza N Tadmor T Sabag A Matarasso Greenfeld S Levy I. The involvement of LAG3+ Plasma cells in the development of multiple myeloma. Blood (2022) 140(Supplement 1):7112–3. doi: 10.1182/blood-2022-160432 PMC10778841 38203720

[100] Bae J Accardi F Hideshima T Tai Y-T Prabhala R Shambley A. Targeting LAG3/GAL-3 to overcome immunosuppression and enhance anti-tumor immune responses in multiple myeloma. Leukemia (2022) 36(1):138–54. doi: 10.1038/s41375-021-01301-6 PMC8727303 34290359

[101] Cho HJ Richard S Lesokhin A Biran N Paul B Vij R. Abstract CT262: Durable responses following anti-TIGIT (BMS-986207) and anti-LAG3 (BMS-980616) in combination with pomalidomide in relapsed myeloma: MMRF MyCheckpoint trial. Cancer Res (2023) 83(8_Supplement):CT262–CT. doi: 10.1158/1538-7445.AM2023-CT262

[102] Corsale AM Shekarkar Azgomi M Plano F Di Simone M Perez C Picone C. High TIM-3 expression may contribute to the functional impairment of bone marrow and circulating gamma delta T lymphocytes during the progression of multiple myeloma. Blood (2022) 140(Supplement 1):9943–4. doi: 10.1182/blood-2022-167131

[103] Jiang W Li F Jiang Y Li S Liu X Xu Y. Tim-3 blockade elicits potent anti-multiple myeloma immunity of natural killer cells. Front Oncol (2022) 12:739976. doi: 10.3389/fonc.2022.739976 35280800 PMC8913933

[104] Sun J Muz B Alhallak K Markovic M Gurley S Wang Z. Targeting CD47 as a novel immunotherapy for multiple myeloma. Cancers (Basel) (2020) 12(2):305. doi: 10.3390/cancers12020305 32012878 PMC7072283

[105] Zhang Y Sheng Z Xia J Ye C Liu Y Wang Z. Exploration of the therapeutic effects of CD47 and CD38 antibody combination in relapsed or refractory multiple myeloma (rrMM) and the correlation with CD47 and CD38 expression. Blood (2022) 140(Supplement 1):9937–8. doi: 10.1182/blood-2022-164755

[106] Abaza Y Zeidan AM. Immune checkpoint inhibition in acute myeloid leukemia and myelodysplastic syndromes. Cells (2022) 11(14):2249. doi: 10.3390/cells11142249 35883692 PMC9318025

[107] Bashey A Medina B Corringham S Pasek M Carrier E Vrooman L. CTLA4 blockade with ipilimumab to treat relapse of Malignancy after allogeneic hematopoietic cell transplantation. Blood (2009) 113(7):1581–8. doi: 10.1182/blood-2008-07-168468 PMC2644086 18974373

[108] Davids MS Kim HT Bachireddy P Costello C Liguori R Savell A. Ipilimumab for patients with relapse after allogeneic transplantation. N Engl J Med (2016) 375(2):143–53. doi: 10.1056/NEJMoa1601202 PMC5149459 27410923

[109] Vago L Gojo I. Immune escape and immunotherapy of acute myeloid leukemia. J Clin Invest (2020) 130(4):1552–64. doi: 10.1172/JCI129204 PMC7108895 32235097

[110] Zeidan AM Knaus HA Robinson TM Towlerton AMH Warren EH Zeidner JF. A multi-center phase I trial of ipilimumab in patients with myelodysplastic syndromes following hypomethylating agent failure. Clin Cancer Res (2018) 24(15):3519–27. doi: 10.1158/1078-0432.CCR-17-3763 PMC6680246 29716921

[111] Garcia JS Flamand Y Penter L Keng M Tomlinson BK Mendez LM. Ipilimumab plus decitabine for patients with MDS or AML in posttransplant or transplant-naïve settings. Blood (2023) 141(15):1884–8. doi: 10.1182/blood.2022017686 PMC10122101 36332187

[112] Penter L Liu Y Wolff JO Yang L Taing L Jhaveri A. Mechanisms of response and resistance to combined decitabine and ipilimumab for advanced myeloid disease. Blood (2023) 141(15):1817–30. doi: 10.1182/blood.2022018246 PMC10122106 36706355

[113] Gojo I Stuart RK Webster J Blackford A Varela JC Morrow J. Multi-center phase 2 study of pembroluzimab (Pembro) and azacitidine (AZA) in patients with relapsed/refractory acute myeloid leukemia (AML) and in newly diagnosed (≥65 years) AML patients. Blood (2019) 134(Supplement_1):832–. doi: 10.1182/blood-2019-127345

[114] Goswami M Gui G Dillon LW Lindblad KE Thompson J Valdez J. Pembrolizumab and decitabine for refractory or relapsed acute myeloid leukemia. J Immunother Cancer (2022) 10(1). doi: 10.1136/jitc-2021-003392 PMC8753450 35017151

[115] Zeidner JF Vincent BG Ivanova A Moore D McKinnon KP Wilkinson AD. Phase II trial of pembrolizumab after high-dose cytarabine in relapsed/refractory acute myeloid leukemia. Blood Cancer Discov (2021) 2(6):616–29. doi: 10.1158/2643-3230.BCD-21-0070 PMC8580622 34778801

[116] Garcia-Manero G Ribrag V Zhang Y Farooqui M Marinello P Smith BD. Pembrolizumab for myelodysplastic syndromes after failure of hypomethylating agents in the phase 1b KEYNOTE-013 study. Leuk Lymphoma (2022) 63(7):1660–8. doi: 10.1080/10428194.2022.2034155 35244520

[117] Chien KS Kim K Nogueras-Gonzalez GM Borthakur G Naqvi K Daver NG. Phase II study of azacitidine with pembrolizumab in patients with intermediate-1 or higher-risk myelodysplastic syndrome. Br J Haematol (2021) 195(3):378–87. doi: 10.1111/bjh.17689 34340254

[118] Daver N Garcia-Manero G Basu S Boddu PC Alfayez M Cortes JE. Efficacy, safety, and biomarkers of response to azacitidine and nivolumab in relapsed/refractory acute myeloid leukemia: A nonrandomized, open-label, phase II study. Cancer Discov (2019) 9(3):370–83. doi: 10.1158/2159-8290.CD-18-0774 PMC6397669 30409776

[119] Daver NG Garcia-Manero G Konopleva MY Alfayez M Pemmaraju N Kadia TM. Azacitidine (AZA) with nivolumab (Nivo), and AZA with nivo + Ipilimumab (Ipi) in relapsed/refractory acute myeloid leukemia: A non-randomized, prospective, phase 2 study. Blood (2019) 134(Supplement_1):830–. doi: 10.1182/blood-2019-131494

[120] Ravandi F Assi R Daver N Benton CB Kadia T Thompson PA. Idarubicin, cytarabine, and nivolumab in patients with newly diagnosed acute myeloid leukaemia or high-risk myelodysplastic syndrome: a single-arm, phase 2 study. Lancet Haematol (2019) 6(9):e480–e8. doi: 10.1016/S2352-3026(19)30114-0 PMC6778960 31400961

[121] Yang X Ma L Zhang X Huang L Wei J. Targeting PD-1/PD-L1 pathway in myelodysplastic syndromes and acute myeloid leukemia. Exp Hematol Oncol (2022) 11(1):11. doi: 10.1186/s40164-022-00263-4 35236415 PMC8889667

[122] Gómez-Llobell M Peleteiro Raíndo A Climent Medina J Gómez Centurión I Mosquera Orgueira A. Immune checkpoint inhibitors in acute myeloid leukemia: A meta-analysis. Front Oncol (2022) 12. doi: 10.3389/fonc.2022.882531 PMC9069679 35530329

[123] Jan M Chao MP Cha AC Alizadeh AA Gentles AJ Weissman IL. Prospective separation of normal and leukemic stem cells based on differential expression of TIM3, a human acute myeloid leukemia stem cell marker. Proc Natl Acad Sci USA (2011) 108(12):5009–14. doi: 10.1073/pnas.1100551108 PMC3064328 21383193

[124] Das M Zhu C Kuchroo VK. Tim-3 and its role in regulating anti-tumor immunity. Immunol Rev (2017) 276(1):97–111. doi: 10.1111/imr.12520 28258697 PMC5512889

[125] Li C Chen X Yu X Zhu Y Ma C Xia R. Tim-3 is highly expressed in T cells in acute myeloid leukemia and associated with clinicopathological prognostic stratification. Int J Clin Exp Pathol (2014) 7(10):6880–8. PMC4230106 25400771

[126] Brunner AM Esteve J Porkka K Knapper S Traer E Scholl S. Efficacy and safety of sabatolimab (MBG453) in combination with hypomethylating agents (HMAs) in patients (Pts) with very high/high-risk myelodysplastic syndrome (vHR/HR-MDS) and acute myeloid leukemia (AML): final analysis from a phase ib study. Blood (2021) 138(Supplement 1):244–. doi: 10.1182/blood-2021-146039

[127] Garcia-Manero G Wei AH Porkka K Knapper S Traer E Scholl S. MDS-420: sabatolimab plus hypomethylating agents (HMAs) in patients with high-/very high-risk myelodysplastic syndrome (HR/vHR-MDS) and newly diagnosed acute myeloid leukemia (ND-AML): subgroup analysis of a phase 1 study. Clin Lymphoma Myeloma Leukemia (2021) 21:S350. doi: 10.1016/S2152-2650(21)01811-5

[128] Majeti R Chao MP Alizadeh AA Pang WW Jaiswal S Gibbs KD Jr.. CD47 is an adverse prognostic factor and therapeutic antibody target on human acute myeloid leukemia stem cells. Cell (2009) 138(2):286–99. doi: 10.1016/j.cell.2009.05.045 PMC2726837 19632179

[129] Liu J Wang L Zhao F Tseng S Narayanan C Shura L. Pre-clinical development of a humanized anti-CD47 antibody with anti-cancer therapeutic potential. PloS One (2015) 10(9):e0137345. doi: 10.1371/journal.pone.0137345 26390038 PMC4577081

[130] Sikic BI Lakhani N Patnaik A Shah SA Chandana SR Rasco D. First-in-human, first-in-class phase I trial of the anti-CD47 antibody hu5F9-G4 in patients with advanced cancers. J Clin Oncol (2019) 37(12):946–53. doi: 10.1200/JCO.18.02018 PMC7186585 30811285

[131] Sallman DA Asch AS Al Malki MM Lee DJ Donnellan WB Marcucci G. The first-in-class anti-CD47 antibody magrolimab (5F9) in combination with azacitidine is effective in MDS and AML patients: ongoing phase 1b results. Blood (2019) 134:569. doi: 10.1182/blood-2019-126271

[132] Sallman D Asch A Kambhampati S Malki MA Zeidner J Donnellan W. AML-196: the first-in-class anti-CD47 antibody magrolimab in combination with azacitidine is well tolerated and effective in AML patients: phase 1b results. Clin Lymphoma Myeloma Leukemia (2021) 21:S290. doi: 10.1016/S2152-2650(21)01694-3

[133] Kim TM Lakhani N Gainor J Kamdar M Fanning P Squifflet P. A phase 1 study of ALX148, a CD47 blocker, in combination with rituximab in patients with non-hodgkin lymphoma. Blood (2019) 134(Supplement_1):1953–. doi: 10.1182/blood-2019-123219

[134] Kasamon YL de Claro RA Wang Y Shen YL Farrell AT Pazdur R. FDA approval summary: nivolumab for the treatment of relapsed or progressive classical hodgkin lymphoma. Oncologist (2017) 22(5):585–91. doi: 10.1634/theoncologist.2017-0004 PMC5423515 28438889

[135] Dada R Usman B. Allogeneic hematopoietic stem cell transplantation in r/r Hodgkin lymphoma after treatment with checkpoint inhibitors: Feasibility and safety. Eur J Haematol (2019) 102(2):150–6. doi: 10.1111/ejh.13186 30341987

[136] Ijaz A Khan AY Malik SU Faridi W Fraz MA Usman M. Significant Risk of Graft-versus-Host Disease with Exposure to Checkpoint Inhibitors before and after Allogeneic Transplantation. Biol Blood Marrow Transplant (2019) 25(1):94–9. doi: 10.1016/j.bbmt.2018.08.028 PMC6310648 30195074

[137] Schoch LK Cooke KR Wagner-Johnston ND Gojo I Swinnen LJ Imus P. Immune checkpoint inhibitors as a bridge to allogeneic transplantation with posttransplant cyclophosphamide. Blood Adv (2018) 2(17):2226–9. doi: 10.1182/bloodadvances.2018019208 PMC6134225 30190282

[138] Saberian C Abdel-Wahab N Abudayyeh A Rafei H Joseph J Rondon G. Post-transplantation cyclophosphamide reduces the incidence of acute graft-versus-host disease in patients with acute myeloid leukemia/myelodysplastic syndromes who receive immune checkpoint inhibitors after allogeneic hematopoietic stem cell transplantation. J Immunother Cancer (2021) 9(2). doi: 10.1136/jitc-2020-001818 PMC7919586 33637601

[139] Tschernia NP Kumar V Moore DT Vincent BG Coombs CC Van Deventer H. Safety and efficacy of pembrolizumab prior to allogeneic stem cell transplantation for acute myelogenous leukemia. Transplant Cell Ther (2021) 27(12):1021.e1–.e5. doi: 10.1016/j.jtct.2021.08.022 34474164

[140] Paul S Zahurak M Luznik L Ambinder RF Fuchs EJ Bolaños-Meade J. Non-myeloablative allogeneic transplantation with post-transplant cyclophosphamide after immune checkpoint inhibition for classic hodgkin lymphoma: A retrospective cohort study. Biol Blood Marrow Transplant (2020) 26(9):1679–88. doi: 10.1016/j.bbmt.2020.06.012 PMC7486273 32592857

[141] Sharma P Goswami S Raychaudhuri D Siddiqui BA Singh P Nagarajan A. Immune checkpoint therapy-current perspectives and future directions. Cell (2023) 186(8):1652–69. doi: 10.1016/j.cell.2023.03.006 37059068

[142] Ramos-Casals M Brahmer JR Callahan MK Flores-Chávez A Keegan N Khamashta MA. Immune-related adverse events of checkpoint inhibitors. Nat Rev Dis Primers (2020) 6(1):38. doi: 10.1038/s41572-020-0160-6 32382051 PMC9728094

[143] Hradska K Hajek R Jelinek T. Toxicity of immune-checkpoint inhibitors in hematological Malignancies. Front Pharmacol (2021) 12:733890. doi: 10.3389/fphar.2021.733890 34483944 PMC8414817

[144] Wang SJ Dougan SK Dougan M. Immune mechanisms of toxicity from checkpoint inhibitors. Trends Cancer (2023) 9(7):543–53. doi: 10.1016/j.trecan.2023.04.002 PMC10330206 37117135

[145] Sallman DA Al Malki MM Asch AS Wang ES Jurcic JG Bradley TJ. Magrolimab in combination with azacitidine in patients with higher-risk myelodysplastic syndromes: final results of a phase ib study. J Clin Oncol (2023) 41(15):2815–26. doi: 10.1200/JCO.22.01794 PMC10414740 36888930

[146] Maakaron J Asch AS Popplewell LL Collins GP Flinn IW Ghosh N. Magrolimab in combination with rituximab + Chemotherapy in patients with relapsed or refractory (R/R) diffuse large B-cell lymphoma (DLBCL). Blood (2022) 140(Supplement 1):3728–30. doi: 10.1182/blood-2022-167772

[147] Zhang T Lin Y Gao Q. Bispecific antibodies targeting immunomodulatory checkpoints for cancer therapy. Cancer Biol Med (2023) 20(3):181–95. doi: 10.20892/j.issn.2095-3941.2023.0002 PMC10038071 36971124

[148] Geuijen C Tacken P Wang L-C Klooster R van Loo PF Zhou J. A human CD137×PD-L1 bispecific antibody promotes anti-tumor immunity *via* context-dependent T cell costimulation and checkpoint blockade. Nat Commun (2021) 12(1):4445. doi: 10.1038/s41467-021-24767-5 34290245 PMC8295259

[149] Li T Wang X Niu M Wang M Zhou J Wu K. Bispecific antibody targeting TGF-β and PD-L1 for synergistic cancer immunotherapy. Front Immunol (2023) 14. doi: 10.3389/fimmu.2023.1196970 PMC10373067 37520520

[150] Chen S-H Dominik PK Stanfield J Ding S Yang W Kurd N. Dual checkpoint blockade of CD47 and PD-L1 using an affinity-tuned bispecific antibody maximizes antitumor immunity. J ImmunoTher Cancer (2021) 9(10):e003464. doi: 10.1136/jitc-2021-003464 34599020 PMC8488710

[151] Rubio-Pérez L Lázaro-Gorines R Harwood SL Compte M Navarro R Tapia-Galisteo A. A PD-L1/EGFR bispecific antibody combines immune checkpoint blockade and direct anti-cancer action for an enhanced anti-tumor response. Oncoimmunology (2023) 12(1):2205336. doi: 10.1080/2162402x.2023.2205336 37114242 PMC10128431

[152] Cheson BD Bartlett NL LaPlant B Lee HJ Advani RJ Christian B. Brentuximab vedotin plus nivolumab as first-line therapy in older or chemotherapy-ineligible patients with Hodgkin lymphoma (ACCRU): a multicentre, single-arm, phase 2 trial. Lancet Haematol (2020) 7(11):e808–e15. doi: 10.1016/S2352-3026(20)30275-1 33010817

[153] Fuentes-Antrás J Genta S Vijenthira A Siu LL. Antibody drug conjugates: in search of partners of choice. Trends Cancer (2023) 9(4):339–54. doi: 10.1016/j.trecan.2023.01.003 36746689

